# Pakistan’s Recommendations for Optimal Management of diabetes from Primary to Tertiary care level (PROMPT)

**DOI:** 10.12669/pjms.335.13665

**Published:** 2017

**Authors:** A. Samad Shera, Abdul Basit, PROMPT Team

**Affiliations:** 1 A Samad Shera, FRCP. Honorary President (IDF), Secretary General (DAP), Director, WHO Collaborating Centre. Diabetic Association of Pakistan; 2 Prof. Abdul Basit, FRCP. Department of Medicine, Baqai Institute of Diabetology and Endocrinology, Baqai Medical University, Karachi, Pakistan; 3 PROMPT Team, FRCP. Department of Medicine, Baqai Institute of Diabetology and Endocrinology, Baqai Medical University, Karachi, Pakistan

**Keywords:** Diabetes guidelines, PROMPT, Referral criteria

## Abstract

Pakistan is a developing country with limited resources and diverse socio-economic standards. Pakistan has high prevalence of diabetes and its complications, which is a great challenge to the existing health care system. National action plans for control of diabetes have been developed and initiatives have been taken but not at an ideal pace. First National Practice Guidelines for Pakistan were published in 1999. It was very helpful in standardizing the management of Type-2 diabetes. In view of important developments in the field of diabetes during the recent years, it was felt that 1999 National Clinical Practice Guidelines edited, should be revised. Also with rapidly increasing number of diabetic patients and the escalating burden on health economy, it is essential to develop a primary to secondary / tertiary care referral system. These guidelines are developed after an extensive research and cover many aspects of diabetes management. This special communication is an extract of a PROMPT document that has already been published as a Supplement in Pakistan Journal of Medical Sciences in 2017. We hope that these guidelines will help in improving the diabetes care in Pakistan.

Pakistan faces many health challenges regarding diabetes due to its high prevalence and related complications. Baqai Institute of Diabetology & Endocrinology (BIDE) in collaboration with Diabetic Association of Pakistan (DAP) recently developed the revised National Clinical Practice Guidelines “Pakistan’s Recommendations for Optimal Management of diabetes from Primary to Tertiary care level”(PROMPT). The main objective of this document is development of the National guidelines for the management of Type-2 diabetes in resource constrained settings in Pakistan and are based on available local and regional evidence. These include special considerations to affordability and availability of medicines and the consensus statements by the Advisory Board for the Care of Diabetes (ABCD). These guidelines not only concentrate on diagnosis and management of diabetes but also provide a key to establish a referral system from primary to secondary and tertiary care and vice versa. Special emphasis has been laid to develop the concept of multi-disciplinary team for the management of diabetes; hence recommendations on nutrition, physical exercise and diabetes education have been included. These recommendations will be revised every two years. Any major changes in the intervening period will be included as addendum/corrigendum. Type-1 diabetes guidelines will be developed after the first inference from Type-1 registry (Diabetes Registry of Pakistan DROP) by end of 2017. The initial step has already been taken for DROP-1 and the registry has been commenced in nearly fifty centers all over Pakistan. Ramadan and diabetes guidelines have been developed by the IDF Diabetes and Ramadan Alliance (DAR Alliance) and Regional guidelines with customization of the available DAR guidelines for national adaptation and dissemination will follow as soon as more comprehensive data is available.

Diabetic foot guidelines will be developed by Pakistan Working Group on Diabetic Foot (PWGDF) in accordance with the International Working Group on the Diabetic Foot (IWGDF) guidance document which has been developed in May 2015 with active participation of faculty of BIDE, Karachi, Pakistan. Gestational diabetes mellitus (GDM) guidelines are being developed by the GDM advocacy board, after the completion of two major projects screening around 25000 pregnant women, the outcomes of these projects are expected to be available by 2018. The main PROMPT document is available online at the website of Pakistan Journal of Medical sciences www.pjms.com.pk/public/site/images/shaukat/BIDE-Prompt-Book.pdf. The salient feature of this document is being given here.

## DIAGNOSTIC CRITERIA

The diagnostic criteria for Type-2 diabetes are fasting plasma glucose: ≥126 mg/dl (≥ 7.0 mmol/dl), random plasma glucose: ≥ 200mg/dl (11.1mmol/l) and plasma glucose after 2 hours of ingesting 75 gm glucose load ≥ 200mg/dl (11.1mmol/l).^1^ International bodies are now recommending HbA1c as diagnostic criteria but national and regional studies are required before it can be included in national guidelines The results from National Diabetes Survey (NDS-2016-17) having more than 10500 sample sizes from all four provinces of Pakistan, expected to be published by the end of 2017, will further address the validity of HbA1c as diagnostic criterion. Population based screening for diabetes may be done using Risk Assessment of Pakistani Individuals for Diabetes (RAPID) scoring system^2^ (Table-I). Glycemic targets categorized for patients with and without complications are shown in Table-II.

**Table-I:** RAPID (Risk Assessment of Pakistani Individuals for Diabetes).

*Risk Factors*	Score**
Age 40-50	1
Age > 50	3
Waist circumference > cut offs*	2
Family History of Diabetes	1

* cut offs are >80 cm in women and >90 cm in men.

** people scoring 4 or more than 4 points should have biochemical tests.

**Table-II:** Glycemic targets for people with Type-2 diabetes^3^.

*Sub Category*	*Fasting blood sugar (FBS) mg/dl*	*Random blood sugar (RBS) mg/dl*	*Bed time sugar mg/dl*	*HbA1c %*
Patients without complications	80-120	80-160	100-140	6.5-7.0
Patients with CCF*, CKD†, CLD‡, Autonomic Neuropathy	80-160	120-180	120-180	7.0-7.5

* Congestive Cardiac Failure,

† Chronic Kidney disease,

‡ Chronic Liver disease.

## REFERRAL CRITERIA

For referral criteria, primary, secondary and tertiary care has been defined.

Primary physician is the first level of contact for individuals, families and communities, in the health care system. Primary health care facility for people with diabetes shall preferably be offered by certified diabetes doctors and educators. The secondary care comprises of multidisciplinary team supervised by a physician having postgraduate qualification or specialized training in diabetes care. The team includes qualified diabetes educators and diabetic foot care assistants. Tertiary care level is a university based teaching hospital comprising of an outpatient and inpatient integrated care along with research and education programs. Routine integrated care involves the patient, physician with special interest in diabetes, clinical nurse specialist or educators trained in diabetes, dietitians, diabetic foot care assistants and/or podiatrists. In all levels of care, proper record maintenance for all treated diabetic patients is advisable.

In primary care level, screening for complications of diabetes should be done at first visit and should be repeated at appropriate intervals, depending upon the progression of disease and include eye examination, foot examination, evaluation of other chronic illnesses plus urine analysis and serum creatinine.^4^ If proteinuria is present; other causes like urinary tract infection, renal calculi, recent fever or exercise etc. should be excluded. If it is negative, test for urinary microalbuminuria is recommended (urinary dipstick for micral test). In case of presence of macro-albuminuria, referral should be made to secondary care for further evaluation. Diabetic patients with other chronic illnesses like tuberculosis, leprosy, hepatitis B or C, cardiovascular disease and secondary hypertension etc., should be directed towards secondary/tertiary care for comprehensive management. Chronic kidney disease, eye emergencies, foot ulcers, and other chronic illnesses ideally shall be treated and managed in secondary care level.^5^ Patients with ulcers not responding to extensive management or showing signs of deterioration at any stage and the subjects for vascular surgery or amputation should be referred to a specialized tertiary care foot clinic. Tertiary care level ideally should have necessary disciplines available such as, cardiology, nephrology, ophthalmology, dentistry, psychiatry, orthopedic surgery, vascular surgery, gynecology and obstetrics etc., providing care for all aspects of diabetes and its complications from prevention to rehabilitation. Any condition that requires more specific intervention should be directed towards more specialized centers.

## NON-PHARMACOLOGICAL MANAGEMENT

### Lifestyle management (LSM)

This section highlights the importance of lifestyle management of diabetes and discusses the goals of nutrition therapy, weight management, physical activity and self monitoring of blood glucose (SMBG). The continuing management of diabetes requires the person living with it to self-manage and be able to make simple decisions regarding meals, exercise and medications. The required frequency of SMBGS should be advised in accordance to the scenarios discussed in BRIGHT recommendations.^3^

### Pharmacological Management

**Fig.1:**
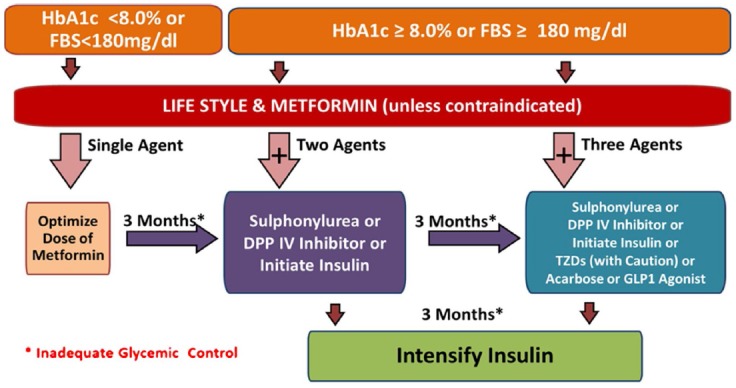
Pharmacological management of diabetes.

Metformin should be prescribed to all patients along with lifestyle modifications, irrespective of their baseline BMI, if there is no contraindications.^6^ Most commonly reported side effects anorexia, nausea, diarrhea and metallic taste can be minimized if metformin is taken with meals. If metformin is contraindicated, sulphonylureas, Dipeptidyl Peptidase IV (DPP4) inhibitors, or insulin can be used as an alternative. Alpha glucosidase inhibitor, glucagon-like peptide 1 (GLP-1), Thiazolidinedione (TZDs) and Repaglinide are also alternate choice of drugs (Fig.1).

**Table-III:** University of Texas diabetic foot ulcer classification system.

*Stages*	*Grade*			

	*0*	*I*	*II*	*III*
A (no infection or ischemia)	Pre- or post- ulcerative lesion completely epithelialized	Superficial wound not involving tendon, capsule, or bone	Wound penetrating to tendon or capsule	Wound penetrating to bone or joint
B	Infection	Infection	Infection	Infection
C	Ischemia	Ischemia	Ischemia	Ischemia
D	Infection and ischemia	Infection and ischemia	Infection and ischemia	Infection and ischemia

### Acute Emergencies

Hypoglycemia and Hyperosmolar Hyperglycemic State (HHS) are two main emergencies encountered in people with Type-2 diabetes. Patients, at the risk of hypoglycemia with the use of medicine, should have proper education on prevention, recognition and its management. For the prevention of repeated hypoglycemia, diabetic patients should have meal or snack containing carbohydrate and protein.^7^ HHS is usually seen in elderly patients and if continues, may result in severe dehydration, which will lead to seizures, coma and eventually death. It must be diagnosed promptly and managed intensively. The goals of treatment are replacement of fluid and electrolyte losses along with the correction of blood glucose to gradually normalize the osmolality and the underlying cause. Causative factors responsible should be identified and corrected to prevent further episodes.

### Microvascular Complications

This section deals with the management of microvascular Complications of diabetes which are categorized as nephropathy, retinopathy and neuropathy. To evaluate nephropathy all people with Type-2 diabetes should be screened annually for microalbuminuria. To evaluate for other causes of renal disease in absence of proteinuria or if kidney disease is rapidly progressive, consider referral to tertiary care. Referral is also considered, if other conditions are present such as anemia, resistant hypertension, electrolyte imbalance, any bone disease or secondary hyperparathyroidism. Detailed history should be taken for any existing eye problem and peripheral neuropathy in all people with Type-2 diabetes. Fundoscopy should be done in every patient at least once a year or more frequently if indicated. For the start of insulin, it is preferable to screen for retinopathy. Almost 50% of these patients can be asymptomatic.

### Macrovascular Complications

### Diabetes & Hypertension

For hypertension control ACE inhibitor/ARBs should be considered as initial therapy in patients with diabetes and hypertension.^8^ Multiple-drug therapy including β-blocker, calcium channel blocker or thiazide diuretic may be considered as an additional therapy to achieve blood pressure targets≤140/90mmHg.

### Dyslipidemia

Diabetic patients should have target LDL cholesterol to be lowered by 50 percent of the baseline or less than 100mg/dl. While, in patients with atherosclerotic cardiovascular disease (ASCVD) risk factors LDL cholesterol target <70 mg/dl is considered. If triglyceride is more than 150mg/dl but less than 500mg/dl, strict lifestyle modifications along with glycemic control and statins is recommended.^9^ If triglycerides are ≥ 500mg/dl in fasting than fibrates can be preferred choice. Addition of a non-statin lipid-lowering agent can be recommended when, statin is not tolerated or a particular LDL-C target is not achieved by statin alone. Aspirin can be recommended to patients, if family history of CVD, hypertension, smoking, dyslipidemia, or albuminuria and age more than 50yrs are present. Clopidogrel is an alternate option if there is any contraindication or people have intolerant to aspirin.

### Diabetic Foot

Management strategies regarding diabetic foot are discussed in detail in this section. The neuropathic ulcers with callus and necrotic tissue should be debrided as soon as possible for appropriate assessment. Patients with deep ulcers involving bone or requiring abscess drainage or if an ulcer is identified as purely ischemic, should be referred to tertiary care center, where foot care facilities are available.

Offloading of ulcer site with custom made shoes, debridement and cleaning of all necrotic tissue and antibiotics with good metabolic control are included in the treatment of ulcer. Patients should also be educated about the warning signs, prevention and management of foot problems and daily foot care practices should be emphasized.

### Diabetes & Obesity

People who are obese and do not have diabetes, can reduce risk of developing diabetes by over 50% by losing 5% of body weight along with regular exercise. Phentermine (since 1959) and Orlistat (since 1999) are currently available drugs to decrease obesity. Bariatric surgical procedures are recommended for people with diabetes having morbid obesity.

### Prompt Team

Zahid Miyan, Musarrat Riaz, Asima Khan, Riasat A. Khan, Asher Fawwad, Rubina Hakeem, Asim Bin Zafar, Waheed Iqbal, Jehangir Khan, Irshad Khoso, Qazi Masroor, Sobia Sabir, Zaman Shaikh, Salma Tanveer, Bilal Bin Younus, Jamal Zafar.
